# Impact of Patient Age on Postoperative Short-Term and Long-Term Outcome after Pancreatic Resection of Pancreatic Ductal Adenocarcinoma

**DOI:** 10.3390/cancers14163929

**Published:** 2022-08-15

**Authors:** Danilo Hackner, Mirianna Hobbs, Susanne Merkel, Timo Siepmann, Christian Krautz, Georg F. Weber, Robert Grützmann, Maximilian Brunner

**Affiliations:** 1 Department of General and Visceral Surgery, Friedrich-Alexander-University (FAU) Erlangen-Nuremberg, 91054 Erlangen, Germany; 2 Division of Health Care Sciences, Dresden International University, 01067 Dresden, Germany; 3 Department of Neurology, University Hospital Carl Gustav Carus, Technische Universität Dresden, 01069 Dresden, Germany

**Keywords:** pancreatic ductal adenocarcinoma, pancreatic resection, overall survival, disease-free survival, age, elderly

## Abstract

**Simple Summary:**

Pancreatic ductal adenocarcinoma (PDAC) is frequently seen in elderly patients. The aim of our retrospective study was to evaluate the impact of age on postoperative short-term and long-term outcomes in patients undergoing curative pancreatic resection for PDAC. Our data confirm that pancreatic resections can be performed with equal short-term outcomes even in older age. However, patient age significantly influenced the overall and disease-free survival of patients with PDAC undergoing primary resection in curative intent. Therefore, the choice of the optimal therapy concept for each patient should be individualized taking into account the patient’s age.

**Abstract:**

(1) Purpose: to evaluate the impact of age on postoperative short-term and long-term outcomes in patients undergoing curative pancreatic resection for PDAC. (2) Methods: This retrospective single-center study comprised 213 patients who had undergone primary resection of PDAC from January 2000 to December 2018 at the University Hospital of Erlangen, Germany. Patients were stratified according the age into two groups: younger (≤70 years) and older (>70 years) patients. Postoperative outcome and long-term survival were compared between the groups. (3) Results: There were no significant differences regarding inhospital morbidity (58% vs. 67%, *p* = 0.255) or inhospital mortality (2% vs. 7%, *p* = 0.073) between the two groups. The median overall survival (OS) and disease-free survival (DFS) were significantly shorter in elderly patients (OS: 29.2 vs. 17.1 months, *p* < 0.001, respectively; DFS: 14.9 vs. 10.4 months, *p* = 0.034). Multivariate analysis revealed that age was a significant independent prognostic predictor for OS and DFS (HR 2.23, 95% CI 1.58–3.15; *p* < 0.001 for OS and HR 1.62, 95% CI 1.17–2.24; *p* = 0.004 for DFS). (4) Conclusion: patient age significantly influenced overall and disease-free survival in patients with PDAC undergoing primary resection in curative intent.

## 1. Introduction

Pancreatic cancer is the seventh most common global cause of cancer-related deaths, with 4.6% of all cancer mortalities [1,2]. It is more common in elderly patients and mainly occurs after 60 years of age, with the highest incidence in people over 70 years [3,4]. The demographic development of an increasing elderly population predicts over 77.7% growth of pancreatic cancer incidence, leading to a rising mortality rate of over 79.9% between 2018 and 2040 [3,5].

Due to its aggressive nature and silent character, only leading to symptoms rather late in the disease’s progression, most patients are at advanced stages when diagnosed. Despite continuous advances in therapy modalities, the prognosis of PDAC is still moderate. The only curative treatment option remains the surgical resection of the tumor. Improvements in perioperative management and surgical techniques significantly reduced morbidity and mortality in pancreatic surgery [6,7]. Morbidity is in the range of 18–42%, and mortality in the range of 0–10% [8,9,10,11].

Nevertheless, the question arises as to at which age patients can benefit from undergoing pancreatic surgery. Several studies were published showing that patient age should not be used as the sole indicator for not performing surgery, and that comparable morbidity and mortality rates can still be achieved for patients over 80 years of age [4,8,10,12]. Moreover, some studies comparing younger and elderly patients showed no inferiority concerning survival after pancreatic surgery [12,13,14,15,16], while other authors could not confirm these findings [10,17,18,19]. The heterogeneity of these studies was criticized, and prognostically relevant factors such as comorbidities, tumor stage, and nodal and resection status were not consistently considered. Therefore, the impact of age on postoperative and especially long-term outcomes remains controversial.

The primary aim of this study was to evaluate the impact of patient age on postoperative outcome, and overall and disease-free survival in patients undergoing curative pancreatic resection for PDAC.

## 2. Patients and Methods

A total of 213 adult patients who had undergone a primary resection of pancreatic ductal adenocarcinoma (PDAC) at the University Hospital of Erlangen, Germany from January 2000 to December 2018 were included. All patient cases were discussed in an interdisciplinary tumor board, and the pancreatic malignancy was classified as primary resectable on the basis of available diagnostics. Patients receiving neoadjuvant chemo- or radiotherapy were excluded from the study.

Patients’ clinical data were retrieved from the clinical information system. Patients’ pathological and survival data were obtained from the Erlangen Cancer Registry of the Department of Surgery. The TNM classification of malignant tumors, as presented by the Union for International Cancer Control (UICC) (according to the 8th edition from 2017), was used to describe the histopathological details [20]. Morbidity was defined as any deviation from the normal postoperative course, was evaluated by Clavien–Dindo classification, and included minor and major morbidities (Clavien–Dindo I–V) [21]. Major morbidity was defined as Clavien–Dindo III–V. Postoperative pancreatic fistula (POPF), delayed gastric emptying (DGE) and postpancreatectomy hemorrhage (PPH) were defined according to the definitions of the International Study Group of Pancreatic Surgery (ISGPS) [22,23,24]. The rates given in the manuscript always included all three grades (for POPF: biochemical leak, Grades B and C; for DGE and PPH: Grades A–C). The median follow-up time of our cohort was 19.0 months (range, 0–198 months).

The local ethics committee approved this retrospective study (22-165-Br).

### 2.1. Study Design

**Figure 1:**
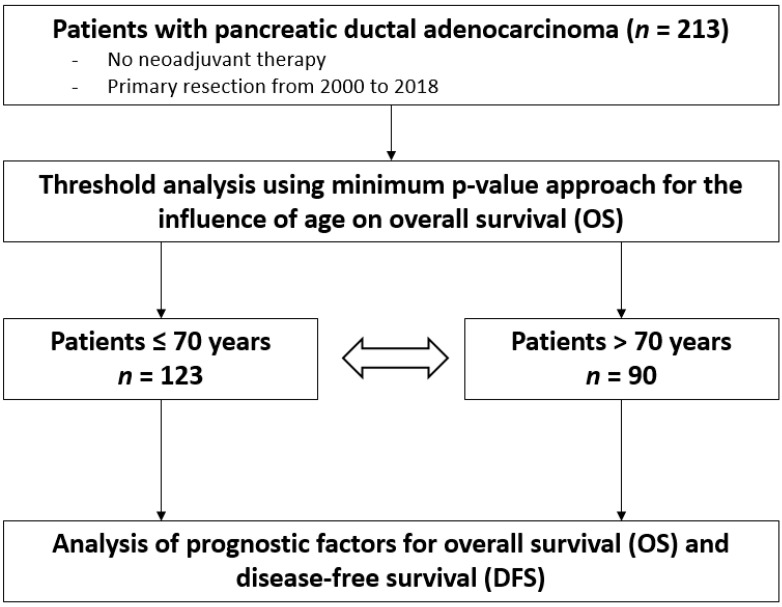
Flowchart of the study design.

We conducted threshold analysis in 5-year intervals using the minimal *p*-value approach for all 213 patients for the influence of age on overall survival (OS) to identify the best cut-off (Supplemental Table S1). On the basis of the identified cut-off of 70 years, patients were stratified into two groups: one group of younger patients (≤70 years, *n* = 123) and one group of older patients (>70 years, *n* = 90). Lastly, demographic, surgical, and short- and long-term outcome data were compared between the two groups (Figure 1).

### 2.2. Surgical Procedures

Visceral surgeons with long-term practical experience in pancreatic surgery performed all surgical procedures, including always an oncological lymphadenectomy. The surgical procedure depended on the tumor localization. Pancreatic head resection was performed as pancreatoduodenectomy either with the resection of the distal stomach (according to Kausch–Whipple operation) or with the preservation of the pylorus (PPPD), depending on the local tumor extent and the individual decision of the surgeon. Pancreatic reconstruction was always performed as pancreatojejunostomy. Pancreatic head resections always included an interaortocaval lymph node dissection. Interaortocaval lymph nodes were evaluated as M1 in the case of tumor involvement, because they do not belong to the locoregional lymph nodes of the pancreas. Therefore, there are some patients classified as pM1, who received primary resection. In the case of intraoperative evidence of liver metastases or peritoneal carcinosis, no resection of the primary tumor was performed. In distal pancreatectomy, the spleen was always removed. In a few cases, a total pancreatectomy was necessary. Additional venous vascular resections and multivisceral resections were performed if necessary for archiving an R0 situation. Arterial vascular resection was only carried out in exceptional cases.

### 2.3. Adjuvant Chemotherapy and Follow-Up

Adjuvant chemotherapy was recommended for all patients except for those with a relevant reduced postoperative general condition. Some patients refused adjuvant chemotherapy. Depending on the patient’s condition, adjuvant chemotherapy was performed with gemcitabine or 5-FU-based chemotherapy. Regular follow-ups were recommended, including quarterly computer tomography (CT) of the thorax and abdomen in the first two years after surgery. From the third year, a CT was recommended for patients every 6 months.

### 2.4. Statistical Analysis

Data analysis was performed with SPSS software (version 28.0, SPSS, Inc., Chicago, IL, USA). Comparisons of metric and ordinal data were calculated with the Student’s *t*-test or Mann–Whitney U test. The chi-squared test was used for categorical data. The minimal *p*-value approach was used to determine the optimal cutoff of age. Overall survival (OS) and disease-free survival (DFS) were calculated for the period between the date of surgery and the date of death or last follow-up, respectively, the period between date of surgery and date of death, the date of local or distant recurrence, or last follow-up. Possible factors related to the patients’ OS and DFS were tested using univariate and multivariate analyses. Variables with a *p* ≤ 0.05 in univariate analysis were used for multivariate analysis with a Cox regression model. Survival curves were plotted using the Kaplan–Meier method and compared with the log-rank test. A *p* value ≤ 0.05 was considered statistically significant.

## 3. Results

### 3.1. Patient Characteristics

The age of the included patients ranged from 45 to 89 years. Most patients were aged between 61 and 70 years (34.7%), followed by 71–80 years (33.8%), 51–60 years (18.3%), over 80 years (8.5%), and equal to or younger than 50 years (4.7%).

The preoperative characteristics of the two patient groups can be found in Table 1. Patients older than 70 years suffered significantly more often from hypertension (69% vs. 46%, *p* = 0.002) and cerebrovascular disease (10% vs. 2%, *p* = 0.031) and were significantly less likely to smoke (3% vs. 37%, *p* < 0.001). Moreover, median albumin level was significantly lower in patients older than 70 years (39.7 g/L vs. 41.2 g/L, *p* = 0.021). Gender, ASA score, BMI, alcohol abuse, comorbidities other than hypertension and cerebrovascular disease, preoperative biliary stenting and preoperative blood values other than albumin, including tumor markers, did not differ between the groups (Table 1).

### 3.2. Surgical and Histopathological Details

Most patients received a pancreatic head resection (76%) followed by distal pancreatectomy (21%) and total pancreatectomy (3%). Additional vascular resection and multivisceral resection were performed in 28% and 18%, respectively. R0 resection was achieved in 87% of the patients.

The surgical and histopathological details, including the TNM stage and the R status of the patients, were similar between the two groups (see Table 2).

### 3.3. Short-Term Postoperative Outcome Parameters

Postoperative outcome parameters are shown in Table 3. Regarding inhospital morbidity, including POPF, DGE and PPH, and inhospital mortality, there was no significant difference between the different age groups. Patients aged above 70 years needed longer postoperative stay (21 vs. 17 days, *p* < 0.001). Of the patients, 52% received adjuvant chemotherapy with a significantly higher rate in the younger group (60% vs. 46%, *p* = 0.038) (Table 3).

### 3.4. Overall and Disease-Free Survival

**Figure 2:**
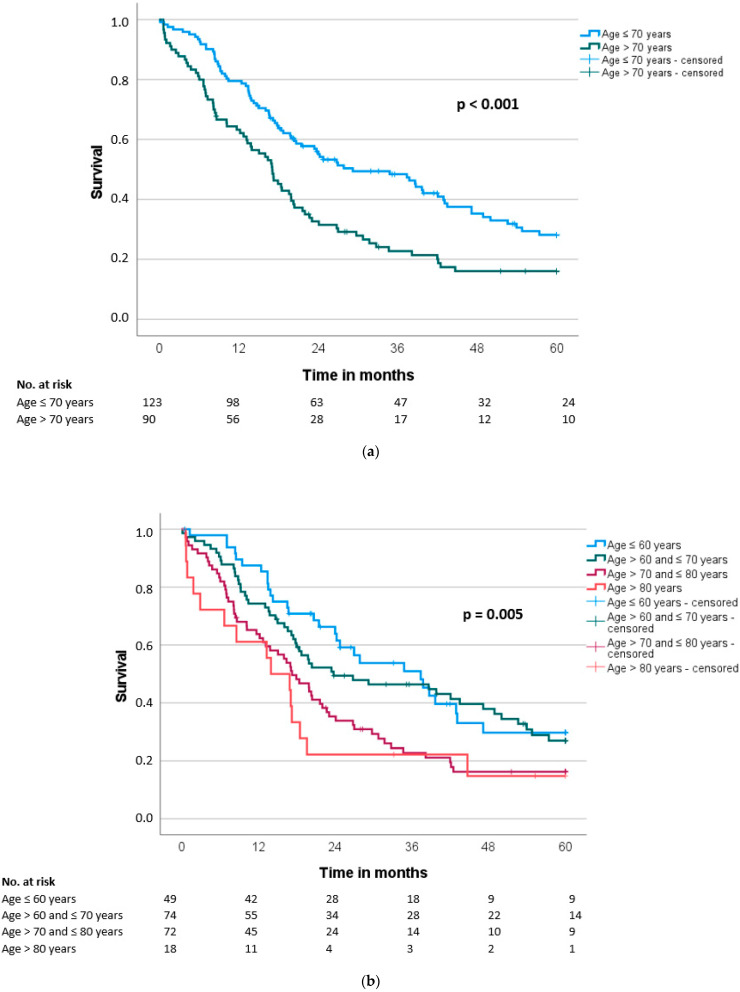
(**a**) Overall survival (OS) according to patients’ age (≤70 vs. >70 years) (*n* = 213). (**b**) Overall survival (OS) according to patients’ age (≤60 vs. >60 and ≤70 vs. >70 and ≤80 vs. >80 years) (*n* = 213).

**Figure 3:**
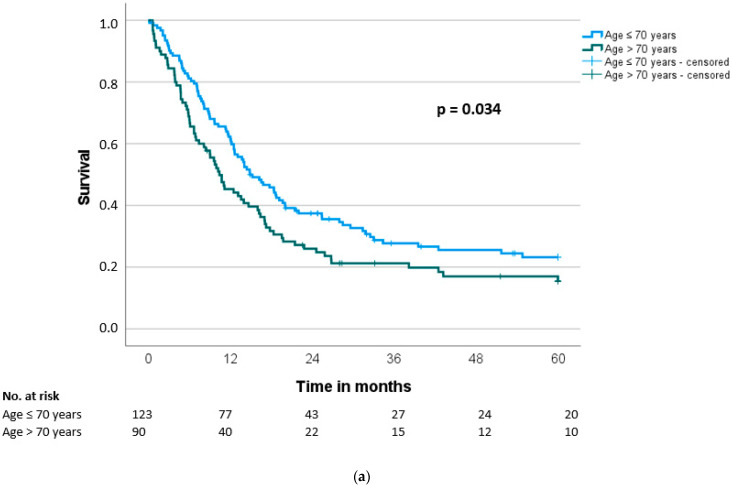

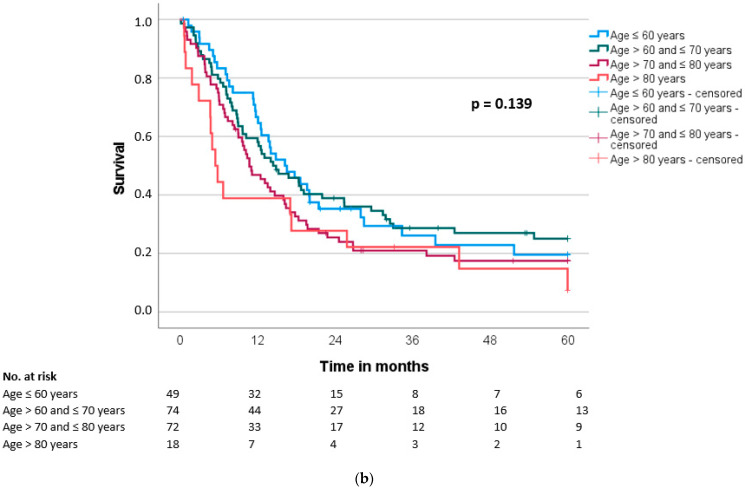
(**a**) Disease-free survival (DFS) according to patients’ age (≤70 vs. >70 years) (*n* = 213). (**b**) Disease-free survival (DFS) according to patients’ age (≤60 vs. >60 and ≤70 vs. >70 and ≤80 vs. >80 years) (*n* = 213).

Median overall survival (OS) and disease-free survival (DFS) were 20.7 ± 1.9 and 13.6 ± 1.4 months, respectively. Patients older than 70 years had a significant shorter OS and DFS compared to the younger group (OS: 17.1 vs. 29.2 months, *p* < 0.001; DFS: 10.4 vs. 14.9 months, *p* = 0.034) (Table 3, Figure 2a and Figure 3a). A more detailed stratification of the patients by age shows significantly shorter OS with increasing age (≤60 years: 37.4 months vs. >60 and ≤70 years: 23.8 months vs. >70 and ≤80 years: 17.3 months vs. >80 years: 14.0 months, *p* = 0.005) (Figure 2b). The same detailed stratification of the patients by age did not reach any significance for DFS (≤60 years: 16.2 months vs. >60 and ≤70 years: 14.4 months vs. >70 and ≤80 years: 10.7 months vs. >80 years: 5.5 months, *p* = 0.139) (Figure 3b).

### 3.5. Prognostic Factors for Overall and Disease-Free Survival

The potentially prognostic factors of patients with resected pancreatic carcinoma regarding OS and DFS are presented in Table 4. Multivariate analysis revealed that age (OS: hazard ratio (HR) 1.95 [95% CI 1.58–3.15], *p* < 0.001; DFS: HR 1.62 [1.17–2.24], *p* = 0.004), lymph node metastasis (OS: HR 2.01 [1.39–2.91], *p* < 0.001; DFS: HR 1.88 [1.32–2.69], *p* < 0.001), R status 1 or 2 (OS: HR 2.77 [1.62–4.73], *p* < 0.001; DFS: HR 1.94 [1.22–3.07], *p* = 0.005) and differentiation with a grading of 3 (OS: HR 1.72 [1.19–2.50], *p* = 0.004; DFS: HR 1.65 [1.17–2.34], *p* = 0.005) were significant independent prognostic factors regarding both OS and DFS (Table 4).

## 4. Discussion

Regarding demographic development, an ever-increasing number of older patients are still in good physical condition, even over 80 years of age. Thus, the influence of age on prognosis may be an increasingly important factor in determining the best therapy concepts in patients with pancreatic carcinoma. For example, in 2020, an 80-year-old person in Germany lives an additional average 8.09 years [25].

Our single-center study comparing patients with primary pancreatic resection for PDAC younger and older than 70 years of age shows that overall survival (OS) and disease-free survival (DFS) were significantly worse in the older group compared to patients younger than or 70 years old (OS: 29.2 vs. 17.1 months, *p* < 0.001; in multivariate analysis: HR = 2.23, 95% CI 1.58–3.15; *p* < 0.001/DFS: 14.9 vs. 10.4 months, *p* = 0.034; in multivariate analysis HR = 1.62, 95% CI 1.17–2.24; *p* = 0.004). Additionally, no difference was noted regarding inhospital morbidity (*p* = 0.255) and inhospital mortality (*p* = 0.073).

In accordance with our results, several studies reported that pancreatic resections can be performed with an equal short-term outcome even in older age [12,26,27]. Next to advances in perioperative management, the most important reason for this fact may result from rational patient selection. In our cohort, there was no difference in ASA score or comorbidities except for arterial hypertension and cerebrovascular disease, which may reflect the rational selection of patients, and explains equivalent morbidity and mortality. However, even with adequate selection, older patients require longer convalescence, which may be expressed in longer postoperative stays (17 days for patients under 70 years and 21 days for patients over 70 years, *p* < 0.001).

Until now, there have been divergent results regarding the prognostic relevance of age in patients with pancreatic carcinoma. In recent years, different studies have been published showing no impaired OS in older groups [12,13,14,15,16]. Eguchi et al. analyzed 36,145 patients in the registry of the Japanese Pancreatic Society, comparing patients younger and older than 40 years of age in clinicopathological characteristics. In this analysis, there was no significant difference in OS and DFS regarding patients receiving surgical resection [15]. Comparable results were demonstrated in a recent study of 10,298 patients comparing patients with a surgical resection of pancreatic carcinoma under and over the age of 60 years. However, in the overall study population, the younger patients had improved OS [16].

The French Surgical Association also reported no significantly different 1-, 3-, and 5-year OS in older patients after the pancreatic resection of pancreatic adenocarcinoma. They analyzed the postoperative outcome in 932 patients with resectable pancreatic adenocarcinoma in different age groups. Patients under 70 years of age were used as a control group for 70–79- and over 80-year-old patients who had undergone pancreatic resection in curative intention [14]. Indeed, various studies, especially more recent ones, have found a difference in OS and DSF in younger and older patients after pancreatic resection [10,28,29,30]. Xu et al. evaluated the long-term prognosis of 95 patients older than 70 years with pancreatic adenocarcinoma compared to patients under 70 years of age using propensity score matching. They revealed better OS in younger patients. In their study population, age was not a sole prognostic factor [30]. A retrospective multicenter study from Japan comprised 1401 patients after pancreatic resection and compared younger patients to octogenarians. The results of this analysis showed that completing adjuvant chemotherapy is a prognostic factor in the very elderly, and that patients above 80 years have poorer prognosis for both resectable and borderline resectable tumors [31]. In addition, Kang et al. analyzed 148,080 patients in South Korea with periampullary cancer. They focused on the effectiveness of pancreaticoduodenectomy in elderly patients, and could identify the age as relevant factor attenuating the survival of patients with periampullary cancer [32]. Nevertheless, all authors stated that pancreatic resection in the elderly is a safe and feasible procedure.

There are several potential reasons for the association between age and survival. First, imbalances in prognostic factors such as the patient’s preoperative performance status and pathological stage can explain different outcomes according to age, as also shown by our results of the multivariate analysis. In our collective, histopathological data were very well balanced between the groups. Regarding the ASA score, however, there was a tendency towards a higher ASA score in the group of older patients (*p* = 0.094).

Second, many studies indicated that preoperative poor nutrition status is a negative outcome factor in pancreatic surgery [33,34,35]. It was frequently seen in the elderly when age groups were compared. Using BMI, albumin and hemoglobin levels as surrogate parameters, the younger and elderly patients in our collective showed good preoperative nutritional status. However, the albumin level was significantly reduced in our group of older patients (*p* = 0.021). Moreover, patients with a higher albumin level tended to have better overall survival in univariate analysis, but without significance (*p* = 0.060).

Third, the undertreatment of older patients is another influential factor [36]. Even recent studies in pancreatic cancer patients with primary surgical resection presented lower rates of adjuvant chemotherapy in the elderly [28,37], whereas studies showed that adjuvant chemotherapy is associated with prolonged survival independent of age [38]. The reasons are, therefore, multifactorial, and one must assume that the patient’s general condition was too weak or they refused adjuvant chemotherapy. Thus, the surgeon’s aim should also be to balance the radicality of the operation and operative burden to the best possible allow the patient an adjuvant therapy. In our cohort, older patients also received adjuvant chemotherapy significantly less frequently (*p* = 0.038). In addition, whether chemotherapy was carried out or not, the type of chemotherapy also plays a decisive role [39]. Unfortunately, these data are missing from our analysis, and this is a relevant weakness of this work.

Therefore, our results suggest that the reasons for a poorer prognosis in elderly patients undergoing primary resection for PDAC might be multifactorial, consisting of a combination of more comorbidities such as hypertension and cerebrovascular disease, a lower preoperative albumin level, and a lower rate of adjuvant chemotherapy.

There are several limitations in our study to be considered. First, the presented prospectively recorded data were evaluated retrospectively and collected from a single center over a long period of 18 years. In the last two decades, the therapy and prognosis of pancreatic carcinoma have changed. At the beginning of the study period, adjuvant chemotherapy was not yet standard. In addition, the regimes of chemotherapy have evolved significantly. Moreover, the number of patients in our analysis was limited. All these limitations, especially the long study period, could always lead to relevant bias. Second, the type of adjuvant chemotherapy was not analyzed. Therefore, chemotherapy regimens could have potentially differed significantly between the groups. Third, the data certainly contain a relevant selection bias, since some patients with advanced age were certainly not presented for surgery at our hospital, and the presented patients were also selected with regard to their ability for surgery. Fourth, our study shows data regarding survival without paying attention to the individual’s quality of life. A quality-adjusted life year (QALY) would represent an even better outcome parameter.

## 5. Conclusions

Upfront surgery for pancreatic carcinoma can be performed in patients over 70 years of age without increasing morbidity and mortality. However, patient age significantly influences the overall and disease-free survival in patients with PDAC undergoing primary resection in curative intent. Therefore, the choice of the optimal therapy concept for each patient should be individualized taking into account the patient’s age.

## Figures and Tables

**Table 1:** Characteristics of patients undergoing pancreatic resection for pancreatic ductal adenocarcinoma stratified to age (≤70 years vs. >70 years).

	Age ≤ 70 Years	Age > 70 Years	*p*-Value
Number	123	90	
Age (years), median [range]	63 [45–70]	76 [71–89]	**<0.001**
Gender, *n* (%)			0.782
Female	56 (46)	43 (48)	
Male	67 (54)	47 (52)	
ASA (*n* = 201) *, *n* (%)			0.094
I	3 (3)	1 (1)	
II	78 (68)	47 (55)	
III	34 (30)	38 (44)	
BMI (kg/m^2^), median (IQR)	25.6 (5.5)	25.6 (4.3)	0.968
Alcohol abuse (*n* = 187) *, *n* (%)	61 (57)	41 (51)	0.461
Nicotine abuse (*n* = 208) *, *n* (%)	44 (37)	3 (3)	**<0.001**
Comorbidity, *n* (%)			
Hypertension	56 (46)	62 (69)	**0.002**
Diabetes	33 (27)	25 (28)	1.000
Cardiovascular	11 (9)	15 (17)	0.095
Pulmonary	13 (11)	6 (7)	0.345
Cerebrovascular	3 (2)	9 (10)	**0.031**
Liver disease	7 (6)	9 (10)	0.297
Preoperative biliary stenting, *n* (%)	62 (53)	50 (56)	0.673
Preoperative hemoglobin (g/dL), median (IQR)	13.0 (2.3)	12.7 (2.3)	0.189
Preoperative WBC (10^9^/L), median (IQR)	7.2 (3.9)	6.7 (3.0)	0.308
Preoperative albumin (g/L), median (IQR)	41.2 (7.9)	39.7 (6.0)	**0.021**
Preoperative CRP (mg/L), median (IQR)	7 (19)	5 (12)	0.441
Preoperative CA19-9 (U/mL) (*n* = 191) *, median (IQR)	73 (254)	108 (415)	0.097
Preoperative CEA (ng/mL) (*n* =155) *, median (IQR)	2.3 (3.4)	2.9 (3.0)	0.540

ASA = American Society of Anesthesiologists classification; BMI = body mass index; WBC = white blood cells; CRP = C-reactive protein. * Missing data.

**Table 2:** Surgical and histopathological details of patients undergoing pancreatic resection for pancreatic ductal adenocarcinoma stratified to age (≤70 years vs. >70 years).

	Age ≤ 70 Years (*n* = 123)	Age > 70 Years (*n* = 90)	*p*-Value
Kind of surgery			0.153
Pancreatic head resection	88 (72)	74 (82)	
Distal pancreatectomy	31 (25)	13 (14)	
Total pancreatectomy	4 (3)	3 (3)	
Portal vein resection, *n* (%)	29 (24)	29 (32)	0.212
Arterial resection, *n* (%)	2 (2)	2 (2)	1.000
Multivisceral resection, *n* (%)	20 (16)	18 (20)	0.587
Operative time (min), median (IQR)	269 (97)	292 (111)	0.075
Intraoperative blood loss (ml), median (IQR)	500 (675)	600 (650)	0.712
Intraoperative blood transfusion, *n* (%)	27 (30)	22 (24)	0.439
T category			0.658
pT1	9 (7)	3 (3)	
pT2	22 (18)	15 (17)	
pT3	90 (73)	70 (878)	
pT4	2 (2)	2 (2)	
*n* category			0.888
pN0	49 (40)	37 (41)	
pN+	74 (60)	53 (59)	
M category			0.243
pM0	114 (93)	79 (88)	
pM1	9 (7)	11 (12)	
R status			1.000
R0	107 (87)	79 (88)	
R1	12 (10)	8 (9)	
R2	4 (3)	3 (3)	
Differentation			0.698
G1	3 (2)	2 (2)	
G2	44 (36)	27 (30)	
G3	76 (62)	61 (68)	

**Table 3:** Outcome parameter of patients undergoing pancreatic resection for pancreatic ductal adenocarcinoma stratified to age (≤70 years vs. >70 years).

	Age ≤ 70 Years (*n* = 123)	Age > 70 Years (*n* = 90)	*p*-Value
Morbidity, *n* (%)	72 (58)	60 (67)	0.255
Major morbidity, *n* (%)	30 (24)	30 (33)	0.167
Mortality, *n* (%)	2 (2)	6 (7)	0.073
Reoperation, *n* (%)	10 (8)	9 (10)	0.809
POPF, *n* (%)	28 (23)	13 (14)	0.160
DGE, *n* (%)	31 (25)	34 (38)	0.052
PPH, *n* (%)	1 (1)	0 (0)	1.000
Length of postoperative stay (days), median (IQR)	17 (9)	21 (16)	**<0.001**
Adjuvant chemotherapy, *n* (%)	74 (60)	41 (46)	**0.038**
Overall survival (months), median (SD)	29.2 (6.3)	17.1 (1.6)	**<0.001**
Disease-free survival (months), median (SD)	14.9 (2.2)	10.4 (1.6)	**0.034**

POPF = postoperative pancreatic fistula; DGE = delayed gastric emptying; PPH = postpancreatectomy hemorrhage; SD = Standard Deviation.

**Table 4:** Prognostic factors of patients with resected pancreatic ductal adenocarcinoma for overall survival (OS) and disease-free survival (DFS).

		Overall Survival (OS)					Disease-Free Survival (DFS)				
		Univariate		Multivariate			Univariate		Multivariate		
	*n*	Median	*p*	HR	95% CI	*p*	Median	*p*	HR	95% CI	*p*
Age ≤70 years >70 years	123 90	29.2 17.1	**<0.001**	**2.23**	**1.58–3.15**	**<0.001**	14.9 10.4	**0.034**	**1.62**	**1.17–2.24**	**0.004**
Gender Female Male	99 114	26.8 19.8	0.119				13.9 12.6	0.276			
ASA (*n* = 201) * I/II III	129 72	24.1 16.9	**0.020**	1.36	0.96–1.91	0.082	14.4 10.2	**0.037**	1.28	0.92–1.77	0.140
Arterial hypertension Yes No	118 95	18.5 24.2	0.193				12.5 16.0	0.251			
Cerebrovascular disease Yes No	12 201	8.2 21.5	0.230				4.7 13.9	0.392			
Preoperative albumin <40 g/L ≥40 g/L	100 113	18.8 26.9	0.060				11.3 16.2	0.131			
Ca19-9 (*n* = 191) * <50 U/mL ≥50 U/ml	72 119	24.2 20.7	0.173				16.5 12.2	0.086			
Kind of surgery PHR DP TP	162 44 7	23.4 17.3 6.7	0.153				14.8 10.2 6.7	0.206			
Vascular resection Yes No	59 154	17.2 22.0	0.242				9.6 14.0	0.286			
Multivisceral resection Yes No	38 175	17.0 23.4	0.111				8.9 14.0	0.104			
T category pT1/pT2 pT3/pT4	49 164	37.8 18.4	**0.013**	1.28	0.82–2.01	0.279	20.0 11.7	**0.006**	1.27	0.84–1.93	0.263
N category pN0 pN+	86 127	39.7 17.8	**<0.001**	**2.01**	**1.39–2.91**	**<0.001**	18.7 11.4	**<0.001**	**1.88**	**1.32–2.69**	**<0.001**
M category M0 pM1	193 20	23.1 12.4	**0.010**	0.81	0.43–1.55	0.532	14.0 8.1	0.077			
R status R0 R1/R2	186 27	23.8 8.8	**<0.001**	**2.77**	**1.62–4.73**	**<0.001**	14.7 8.7	**0.006**	**1.94**	**1.22–3.07**	**0.005**
Differentiation G1/G2 G3	76 137	37.8 17.1	**<0.001**	**1.72**	**1.19–2.50**	**0.004**	19.7 11.7	**<0.001**	**1.65**	**1.17–2.34**	**0.005**
Morbidity Yes No	132 81	19.8 29.2	0.376				13.1 13.9	0.820			
Reoperation Yes No	19 192	17.0 22.0	0.131				14.0 13.7	0.400			
Adjuvant chemotherapy Yes No	115 98	23.4 17.2	0.297				14.8 11.0	0.557			

ASA = American Society of Anesthesiologists classification, PHR = Pancreatic head resection, DP = Distal pancreatectomy, TP = Total pancreatectomy. * Missing data.

## Data Availability

The data presented in this study are available upon reasonable request from the corresponding author.

## Supplementary Materials

The following supporting information can be downloaded at: https://www.mdpi.com/article/10.3390/cancers14163929/s1. Table S1: minimal *p*-value approach for influence of age on overall survival (OS).
