# Supplementary material for: Impact of Patient Age on Postoperative Short-Term and Long-Term Outcome after Pancreatic Resection of Pancreatic Ductal Adenocarcinoma
Source: Cancers (Basel). 2022 Aug 15;14(16):3929. doi: 10.3390/cancers14163929 (PMC9406071; doi:10.3390/cancers14163929)
Supplement: Supplementary file 1 [file cancers-14-03929-s001.zip › cancers-1841666-supplementary.pdf]

Sup. Table S1: Minimum p-value approach for influence of age on overall survival (OS)

		Lower age		Higher age	
**Evaluated cut-off (age (years))**	***p*-value**	**n**	**Overall survival (OS) (months), median (SD)**	**n**	**Overall survival (OS) (months), median (SD)**
≤ 60	0.041	49	37.4 (6.5)	164	18.5 (1.2)
≤ 65	0.004	82	37.8 (6.3)	131	18.0 (1.1)
≤ 70	< 0.001	123	29.2 (6.3)	90	17.1 (1.6)
≤ 75	0.005	159	24.0 (3.3)	54	16.4 (2.3)
≤ 80	0.051	195	22.7 (2.3)	18	14.0 (3.8)

SD = Standard Deviation.
